# “There’s no billing code for empathy” - Animated comics remind medical students of empathy: a qualitative study

**DOI:** 10.1186/s12909-016-0724-z

**Published:** 2016-08-12

**Authors:** Pamela Tsao, Catherine H. Yu

**Affiliations:** 1 Department of Medicine, Division of Endocrinology and Metabolism, University of Toronto, 200 Elizabeth Street, EN 12-243, Toronto, ON M5G 2C4 Canada; 2 Department of Medicine, Division of Endocrinology & Metabolism, St. Michael’s Hospital, Li Ka Shing Knowledge Institute of St. Michael’s Hospital, University of Toronto, 30 Bond St, Toronto, ON M5B 1W8 Canada

**Keywords:** Empathy, Medical student education, Comics

## Abstract

**Background:**

Physician empathy is associated with improved diabetes outcomes. However, empathy declines throughout medical school training. This study seeks to describe how comics on diabetes affect learning processes for empathy in medical students.

**Methods:**

All first- or second-year students at a Canadian medical school were invited to provide written reflections on two comics regarding diabetes and participate in a focus group. Responses were analyzed qualitatively for emergent themes. Students completed the Jefferson Scale of Physician Empathy (JSPE) at baseline, after the comic, and after the focus group. Linear mixed model statistical analyses were performed.

**Results:**

Thirteen first-year and 12 second-year students participated. Qualitative analysis revealed four themes: 1) Empathy decline and its barriers; 2) Impact of the comic and focus group on knowledge, attitudes and skills; 3) Role of the comic in the curriculum as a reminder tool of the importance of empathy; 4) Comics as an effective medium. Baseline mean JSPE scores were 116.4 (SD 10.5) and trended up to 117.2 (SD 12.5) and 119.6 (SD 15.2) after viewing the comics and participating in the focus groups, respectively (*p* = 0.08).

**Conclusions:**

Animated comics on diabetes are novel methods of reminding students about empathy by highlighting the patient perspective.

**Electronic supplementary material:**

The online version of this article (doi:10.1186/s12909-016-0724-z) contains supplementary material, which is available to authorized users.

## Background

Empathy is an essential component of patient care with benefits for both the patient and physician. Empathy is defined as an “understanding of patients’ experiences, concerns and perspectives combined with a capacity to communicate this understanding” [1] and then to act with “an intention to help” [1]. Clinical empathy has several advantages. Patient-perceived physician empathy is associated with greater patient satisfaction and compliance [2]. Physician empathy is also associated with improved clinical outcomes in diabetes; for example, patients of physicians with higher empathy scores are more likely to achieve target hemoglobin A1C and LDL-C levels [3]. Empathy is further associated with increased personal well-being in internal medicine residents [4] and is a learning objective of the Association of American Medical Colleges [5].

Despite the recognized importance of empathy, studies show a decline in medical student empathy throughout training. A review of 18 studies on changes in empathy in medical school and residency showed a significant decrease in empathy, particularly in the third year [6]. To date, there is some evidence that empathy can be taught. In a review by Batt-Rawden et al. [7] they identified 18 studies published since 2003, evaluating educational interventions to increase empathy in undergraduate medical students. Fifteen studies reported significant increases in empathy scores with twelve studies using various validated outcome measures (examples include the Jefferson Scale of Physician Empathy (JSPE), empathy tendency scale, empathic skill scale). The review identified several types of educational interventions that may be used, including the creative arts.

Use of humanities in medical education has been well documented. Educators have used literature in the form of novels, short stories, plays and poems to highlight social and cultural aspects of illness [8]. Graphic stories, or comics, are another creative teaching method with recent introduction into medical education. They refer to “the combined use of images and text, sequentially, to tell a story, where the images complement and/or enhance the text” [9]. Comics can serve as an efficient means of communicating complex information. Juxtaposing text and images and changing text sizes and styles can convey a powerful message in very little space [10]. Green et al. found that studying comics can teach observational skills [10]. For example, physicians must gather information from a patient’s history, physical exam, social and cultural context to create a narrative. In the same way, reading comics requires the cognitive task of reconstructing graphic and textual clues and filling in the blanks between panels to form a story. With the above characteristics, comics may serve as a novel method to enhance empathy in medical education.

While teaching with comics may be a new idea, using a medium to facilitate reflection for learning is well recognized in medical education. In Kolb’s experiential learning cycle, there are four phases to learning [11]. The first phase involves having an experience such as a patient encounter, but this is not sufficient for learning. The second phase of reflection and third phase of “abstract conceptualization” are vital to learning by making sense of the experience and recognizing learning needs. The fourth phase of “active experimentation” involves trying new skills and knowledge to plan for the next experience. Comics are particularly well suited for reflection as they allow the reader to pause, consider certain panels and build the narrative at their own pace as opposed to videos or plays [12].

The effect of graphic stories on learning empathy in medical students has not previously been explored. The purpose of our study is to discover how an online animated comic strip series on diabetes management affects learning processes for empathy in medical students. This is primarily a qualitative study to understand the mechanism by which comics can affect empathy. In order to understand which strategies can be used to improve empathy, we also explored barriers to maintaining empathy during medical training, such as those related to medical education itself, students and the training environment. A secondary aspect of the study is to assess quantitative changes in empathy scores related to observing and discussing the comics.

## Methods

### Study design

This was primarily a qualitative study using focus group methodology; secondarily, we assessed for quantitative changes in empathy scores.

### Setting and participants

All medical students at a Canadian university in their first or second years of study (250 students in each year) in the 2012–2013 academic year were eligible to participate. The undergraduate medical curriculum in this program includes 2 years of preclinical studies and 2 years of clinical clerkship. Students in first or second year have 4 h per week of clinical skills teaching and optional observerships in family medicine. This is primarily a study to inform how comics can affect the learning of empathy rather than to show halting of empathy decline. As such, we chose to study first or second year students prior to the recognized empathy decline in the third year [13]. Participants were recruited between March and May of 2013 via institutional listserv, in-person presentation prior to an educational session as well as personal communication and email. We anticipated that a sample size of 4–5 focus groups (5–8 participants per group) would be required to achieve saturation of themes in a homogenous sample of typical cases [14]; however we recruited and conducted interviews through the above methods repeatedly until saturation was achieved.

### Intervention

A needs assessment was previously completed with patients with diabetes to identify their struggles with chronic disease (unpublished). This identified key topics such as challenge of behavior change, burnout, fear of insulin initiation, guilt, denial and frustration with complications. Learning objectives were developed from these key topics to guide the content of the comic strips. Particular use of colour and animation were chosen to align with the learning objectives. Two animated online comic strips on diabetes management were created. The first depicts a patient who is asked to start on insulin and chronicles their fear of insulin initiation [15]. The second shows a patient trying to follow lifestyle recommendations on a daily basis and their resultant self-management burnout [16]. Links to the comic strips were sent to participants via email. Students individually viewed the two comic strips, separated by one week to allow reflection. One week after viewing the second comic, students met in focus groups to reflect on the comics and their impact on empathy. During the focus group, students had access to the comics to refer to them as needed.

### Outcome

The initial purpose of our study was to discover how an online animated comic strip series on diabetes management affects learning processes for empathy in medical students. In the focus groups, we sought to determine the impact of the comics on empathy such as effect on: awareness of the issue, knowledge of patient perspective, observational skills and recognition of non-verbal expressions of affect. We also obtained feedback on the comic as a medium and the characteristics that make it effective or ineffective. Thus, this was initially designed to assess how comics might affect learning processes for empathy. However, during the first focus group session, participants commented on the lack of empathy of the physician in the comic strip as well as their own decline in empathy. This unveiled a rich area of enquiry that we chose to further explore. Given the qualitative nature of the study, the interview guide was revised in an iterative process to further explore this line of enquiry.

A secondary objective of the study was to assess quantitative changes in empathy after viewing the comic strips and after the focus group. We evaluated empathy using the Jefferson Scale of Physician Empathy (JSPE). The JSPE is a self-administered scale with 20 items answered on a 7-point Likert-type scale. The total score ranges from 20 to 140, where higher total scores are associated with greater empathy. The JSPE was created specifically in the context of patient care. There is evidence for its construct validity [17], predictive validity [18], test-retest reliability [18] and internal consistency in medical students with Cronbach’s alpha 0.89 [17].

### Data collection

Baseline demographic information (age, gender, first language, year of study, academic and clinical background, interested specialty, and patient exposure) was collected from all participants via online survey sent by email. Students read a case vignette (Additional file 1) about a patient with diabetes then provided written responses to the following open-ended statements: “1. As his doctor, how do you feel? 2. How do you think Mr. Williams feels?” After viewing each comic, students also provided written responses to the following open-ended statement: “After reading the comic, please provide any comments.” Students’ written responses to the case vignette and to each comic were collected via online survey.

Semi-structured interview guides with open-ended questions were developed after pilot testing in a general population to ensure clarity (Additional file 2). PT, a female resident, conducted the focus group sessions in a private university classroom. We chose to use focus groups because group discussion is a familiar process for pre-clerkship students that reflects the benefits of group learning, and facilitates the sharing of ideas and experiences, important in a study on empathy [19]. A research assistant was present to aid with set up and take field notes but did not interview the participants. PT was not involved in participant medical education or evaluation. As data was analyzed, the interview guide was revised in an iterative process. The focus groups lasted 30 to 45 min. Interviews were digitally audiotaped and transcribed verbatim by PT. Interviews were conducted until saturation of themes. We ascertained whether saturation was achieved through concurrent data collection and analysis. Specifically, we initially recruited and scheduled students for 3 focus group sessions. Upon collecting and analyzing this data and not yet finding saturation (assessed by 2 coders independently), we conducted further recruitment and data collection until no new information was found (2 more cycles) for a total of 5 focus groups.

The JSPE was administered via online survey to participants at baseline, immediately after viewing the two comics and immediately after attending the focus group.

### Data analysis

Focus group transcripts and students’ written responses to the case vignette and comics were read, coded and reviewed independently by the two authors (PT and CHY). A coding framework of emerging themes was derived from the data based on consensus between the two authors using a qualitative descriptive approach ([20] and the constant-comparison method [21]. NVivo10 software was used for analysis. Emergent themes were used to refine the focus group interview guide in an iterative process.

For the quantitative outcome, the mean total JSPE score was calculated separately at baseline, immediately after the second comic and immediately after the focus group. The scores were compared using linear mixed model for repeated measurements for significant differences at *p* ≤ 0.05.

## Results

### Participants

There were 5 focus groups (25 participants) in total. The characteristics of the study participants are available in Additional file 3.

### Qualitative themes

The qualitative data was organized into four main themes: empathy decline and its barriers; the impact of the comic and focus group on knowledge, attitude and skills; the role of the comic in the curriculum as a reminder of empathy; and comics as an effective medium. Overall, students were concerned about the decline in empathy and felt that the comic, a novel medium, served as a reminder tool of the importance of empathy by emphasizing the patient perspective. Representative quotes for each theme appear in Additional file 4.

### Empathy decline and its barriers

#### Students were aware and concerned of empathy decline

Most participants were strikingly aware that empathy declines throughout medical training. Some participants felt that cynicism was an expected part of the culture of medicine (Additional file 4; 1a). Another participant described how troubling this inevitability was and wanted to intervene (Additional file 4; 1a).

#### Barriers to maintaining empathy

The students provided many reasons for why empathy might decline. A prominent barrier that was identified by multiple participants was the emphasis in medical school on the medical expert role rather than the psychosocial aspects (Additional file 4; 1b, i). Other environmental barriers to maintaining empathy included unhealthy environments, lack of financial incentive, no academic consequence to lack of empathy, lack of positive role models and focus on passing examinations (Additional file 4; 1b, ii-vi). Some student barriers included limited time, personal stress and fatigue, lack of social diversity and desensitization (Additional file 4; 1b, vii-x).

#### Strategies to halt empathy decline

While students identified several barriers to maintaining empathy, they also provided strategies to prevent empathy decline. They proposed teaching strategies such as: teaching efficient methods of communication and providing more feedback on empathic skills including patient opinions (Additional file 4; 1c). They also identified ways to maintain empathy in busy clinical settings for example using non-verbal communication, prioritizing their time and validating patient concerns (Additional file 4; 1c).

The statements highlighted show that participants are aware of a decline in empathy and felt this occurred from a variety of reasons. Most strongly, it was due to the persistent culture of cynicism in medicine, the focus on the medical expert role and a lack of time. Despite this, students felt troubled by the decline and identified potential strategies to halt it.

### Impact of the comic and focus group on knowledge, attitude and skills

#### Comics shed light on the patient perspective and on chronic disease management (knowledge)

A strong theme from our data shows that nearly all of the students thought that the comic increased their knowledge of the patient perspective (Additional file 4; 2a, i). One participant revealed that their prior knowledge base about patients with diabetes starting on insulin was in fact opposite to the actual patient perspective depicted in the comic. The comic caused a drastic shift in their knowledge (Additional file 4; 2a, ii). Other participants found that the comic helped them recognize the difference between a patient’s perceived and actual perspective (Additional file 4; 2a, iii). Students identified that significant differences can exist between patient and physician perspectives as well (Additional file 4; 2a, iv). After viewing the comics, participants had increased awareness of the challenges of behavior change and of following a daily routine in a chronic disease such as diabetes (Additional file 4; 2a, v). They also recognized the importance of taking the next steps to motivate patients in self-management after observing strategies used in the comic (Additional file 4; 2a, vi).

The representative quotes show that the comics helped participants increase their knowledge of the patient perspective, sometimes in drastic ways. It also allowed them to recognize the differences between true patient perspective and perceived patient or physician perspectives. The comics further expanded their awareness of the challenges of and strategies used in chronic disease management.

#### Comics emphasized the importance of empathy in patient encounters (attitude)

Our data also revealed that the comics had an impact on participant attitude towards the importance of empathy, specifically the value of understanding the patient perspective (Additional file 4; 2b, i). Some participants explained how the comic would affect their approach in a future patient encounter (Additional file 4; 2b, ii). Actual changes in student attitudes were highlighted in their responses to the case vignette on a patient with diabetes at baseline compared to at the end of the study. Selected quotes are shown in Table 1.

**Table 1:** Response to clinical vignette before and after viewing comics

Question asked: “As his doctor, how do you feel?” Participant 2, female, year 1	
Before	After
“It is a very difficult situation because the patient's BMI and HbA1c are increasing and he has been unable to follow my recommendations.”	“A bit frustrated because the patient did not follow my recommendations… obliged to do my best to understand why the patient found it hard to adhere and work with him to make it easier to manage his diabetes.”

#### Comics enhanced one’s observational skills regarding communication (skills)

Another impact of the comic was that participants used their observational skills when interpreting them. Several students were able to identify various non-verbal communication techniques, both effective and ineffective, which were used in the comic (Additional file 4; 2c, i). Another participant observed the repetitive nature of the second comic in conveying the daily routine and burnout associated with chronic disease (Additional file 4; 2c, ii). They also observed the use of colour changes to convey different moods and interactions (Additional file 4; 2c, iii).

The quotes show that students used their observational skills to identify body language, repetition and colour changes in analyzing the comics.

### Role of the comic in the curriculum as a reminder of empathy

When participants were asked to identify what the comic could be used for, the most common response was its role as a reminder of empathy. Students recognized that they had learned about empathy and communication skills in pre-clerkship studies but that its emphasis had waned. They found that the comic served as a useful and quick tool to remind them of the value of being empathic (Additional file 4; 3a). Another participant highlighted its potential utility during times of stress and burnout to ground oneself and relate back to the patient (Additional file 4; 3b).

The participants had differing ideas of how the comics might be integrated into the curriculum as a reminder tool. These included showing the comics prior to lectures, posting them in hospitals or study areas, e-mailing them to the students, posting them on a social media website, and viewing them in small group sessions such as clinical skills or problem-based learning courses. Some participants felt that the comics should be presented in clerkship rather than pre-clerkship because this is the time when empathy declines (Additional file 4; 3c). They also felt that the comics should be pertinent to the clinical block (Additional file 4; 3d). Finally, some students wanted the comic shown to them at multiple stages as a repeated reminder (Additional file 4; 3e).

Their comments show that participants found the comics to be a useful reminder tool of the importance of empathy with potential integration into the relevant clerkship rotations at repeated stages.

### Comics as an effective medium

Participants enjoyed the use of comics as a medium. They described the comics as novel, humorous, and memorable (Additional file 4; 4a). Several participants also commented that comics were an efficient way of communicating information, particularly in comparison to text (Additional file 4; 4b). Students also found that the visual effects of the comics helped to personify the characters. This allowed them to relate to the characters and imagine what they might be going through (Additional file 4; 4c). Although other creative arts interventions such as films also have a strong visual appeal, participants found that the benefit of comics as a medium was the ability to reflect on the content (Additional file 4; 4d).

Overall, the comments suggest that comics are a novel, effective teaching medium as information can be conveyed efficiently to visually bring emotions to life, while still allowing for personal reflection.

### Quantitative outcome

**Fig. 1:**
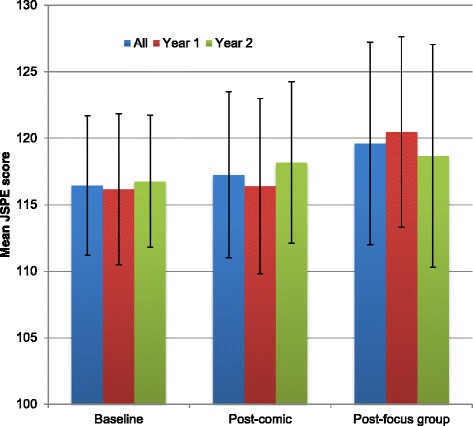
Changes in mean JSPE scores (SD) by group: all, year 1 and year 2

The secondary quantitative outcomes are shown in Fig. 1. JSPE scores trended up over time but were not statistically significant for time (*p* = 0.08), gender (*p* = 0.98) or year of study (*p* = 0.58) effect.

## Discussion

This study of medical students revealed that they are certainly aware of empathy decline over time and recognized several barriers to maintaining empathy. The overwhelming response from the participants was that a major barrier is the focus on medical content rather than psychosocial aspects of medical education. They also identified negative impacts such as unhealthy environments, a culture of cynicism and a lack of positive role models, which have previously been reported as aspects of the curriculum contributing to empathy decline [6]. In fact, a recent study of practicing physicians’ views of influences on their empathy development during their medical education reported that physicians felt that medical education does not promote empathy development [22]. In particular, the lack of role models is troubling given that most of what students learn about communication occurs from observing their teachers [23]. One study examined medical students’ perceptions of how their learning occurred across explicit versus implicit curricula. The explicit curriculum includes learning objectives specified in written course materials. The implicit curriculum is defined as “what is taught through institutional culture and organization as well as through interpersonal interactions with instructors”. The study revealed that learning about sciences and building patient stories (through history, physical exam and procedures) occurred mainly in the explicit curriculum and required a significantly greater amount of their time than other objectives. In contrast, learning about communication and understanding patients predominantly occurred in the implicit curriculum, which depends on interactions with role models [23]. This study and our participants’ views highlight to those involved in medical curriculum development that informal learning environments are crucial to communication skills development. It should also remind staff physicians and supervising residents of the important responsibility they hold as role models.

Participants also identified student barriers to maintaining empathy such as lack of time due to high workload, lack of social diversity and personal stress and fatigue, in line with a review on empathy decline, identifying “distress” as a key factor in the decline [6], and high workload and lack of social support as major contributors to distress. Thus, interventions aimed at improving empathy need to take into account that students already have significant time constraints and personal stress.

To our knowledge, this is the first study to report that students explicitly identified the lack of financial incentive as a barrier to maintaining empathy. The association between payment and empathy is suggested by one German study that found that patients with private health insurance plans rated physician empathy higher compared to those with statutory plans [24]. This appeared to be mediated by physicians spending more time with the patient: physicians with private insurance plans talked more frequently with their patients during a hospital stay compared to those with statutory plans, while spending the same time per visit. The study suggested that financial incentives affect how physicians allocate their time and that time is a crucial factor in providing empathy. Taken together with the barrier of lack of time, this emphasizes that time-efficient methods of teaching empathy using short interventions as with our comic strips are required.

Another novel barrier noted by our participants is the perception that there is no academic consequence to not showing empathy. A previous study has shown that students perceive the psychosocial aspects of medicine to be less important [25]. The study also showed that time devoted to social aspects of medicine at one US medical school declined through the year. For first year students, this decreased from 95 h per month in August to 50 h per month in June. For second year students, this similarly declined from 42 h per month to 16 h per month through the year. They proposed that programs increase the number of formal examinations of empathy and psychosocial aspects of care. However, our participants also voiced their concern that formal examinations may not cultivate true empathy but instead become just a “checkbox” at a station. This has been discussed in the literature noting that standardized patient exams train students to “put on good performances” [26] and promote superficial interviewing [27]. While standardized patient assessments may not be optimal, it may be one way to show students that empathy is valued in the medical teaching community. Positive role modeling, as mentioned above, is another way of showing students by example that empathy is important.

Our study also identified potential strategies to maintain empathic interaction in practice such as validating patient concerns and using non-verbal communication, echoing the literature on communication skills training [28]. However, our participants also proposed that providing actual patient feedback on their empathic skills might improve empathy. While studies on multisource feedback (or 360° evaluation) including patient feedback in medical students [29] and residents [30] exist, these studies examine multisource feedback as an evaluation tool, rather than a teaching tool. Our participants have raised the possibility that patient feedback might also be a teaching tool for empathy, which has not previously been explored.

This study shows that comics can serve as an effective educational tool by helping students gain knowledge, change attitudes towards patient encounters, improve observational skills and reflect on empathy and patient encounters. Reflection is a crucial step in the learning cycle, as outlined in Kolb’s learning theory [11]. In our study, participants have had previous patient encounters (*“concrete experience”*). They then viewed the animated comics at their own pace, responded to questions about the comics and discussed them in a focus group. These steps allowed for *“reflective observation”* and led to making generalizations from their reflections (*“abstract conceptualization”*). For example, some participants identified the importance of empathic communication by viewing the comics and subsequently changed their attitudes and approach to patient encounters. They identified learning needs and skills to be applied in future experiences. The fourth phase of Kolb’s learning cycle (*“active experimentation”*), which involves applying the new knowledge and skills to a future patient encounter was not addressed in our study. Future studies could expose participants to the comic in a clinical setting, such as a diabetes clinic, for active experimentation of their new skills. As participants felt that the comics could be displayed repeatedly over time, they could serve as important reflective aids in iterative cycles of learning.

The main strength of this study was its qualitative nature. Since the use of comics in medical education has not been previously explored, this study was not attempting to verify existing theories. Instead, we sought to describe how medical students might learn about empathy using animated comics. Our iterative design and data collection until saturation ensured richness of data.

Our study has several limitations including small sample size for quantitative analysis and a single university setting. The population may have been self-selected for individuals with higher baseline empathy as the majority of participants were interested in people-oriented specialties. There is some evidence from prior studies that students preferring people-oriented specialties have higher empathy scores [13]. However, our study still had participants with an interest in all major specialties. As well, the mean empathy score at baseline (116.4) is comparable to scores reported in the literature for all first and second year medical students at Jefferson Medical College (115.5 and 115.1 respectively) [1] and Boston University School of Medicine (118.5 and 118.2 respectively) [13]. We did ensure analytic rigor by using multiple analysts [14, 31].

An additional limitation is that the exposure to the comics and responses were collected within a short time; thus we cannot comment on the sustainability of the intervention.

## Conclusion

Medical students are aware of empathy decline and seeking strategies to prevent this decline. Future research and curriculum development should focus on efficient tools and teaching opportunities in informal environments including role modeling and patient feedback. Animated comics on diabetes are one novel and efficient teaching medium that was well received by medical students in this study. The comics may serve as a reminder and reflection tool for the importance of empathy by increasing knowledge on the patient perspective, attitudes towards empathy and observational skills. Future studies on the use of comics should focus on students in clerkship, when empathy is known to decline.

## Additional files

Additional file 1: Case vignette. Description of data: Case vignette used as prompt for reflective questions before and after comic viewing. (PDF 218 kb) Additional file 2: Semi-structured interview guide. First version of semi-structured interview guide used for focus group interviews. (PDF 267 kb) Additional file 3: Table S1. Description of data: Characteristics of the study participants. (PDF 32 kb) Additional file 4: Table S2. Description of data: Themes and representative quotes from qualitative data. (PDF 53 kb)
