# Supplementary material for: “There’s no billing code for empathy” - Animated comics remind medical students of empathy: a qualitative study
Source: BMC Med Educ. 2016 Aug 12;16:204. doi: 10.1186/s12909-016-0724-z (PMC4983096; doi:10.1186/s12909-016-0724-z)
Supplement: Additional file 1: Case vignette. — Description of data: Case vignette used as prompt for reflective questions before and after comic viewing. (PDF 218 kb) [file 12909_2016_724_MOESM1_ESM.pdf]

Supplementary File 1. Case vignette

Mr. Williams is a 55 year-old man with Type 2 diabetes. You are seeing him in follow-up in your clinic. At the last visit 3 months ago, Mr. Williams' BMI was 29.5 and Hemoglobin A1C was 8%. You encouraged him to exercise, lose weight, and monitor his blood sugar regularly. At this visit, Mr. Williams admits that he has not been able to follow any of your recommendations. His BMI is 30 and Hemoglobin A1C is 9%. You again ask him to adhere to the non-pharmacologic recommendations and decide to start him on insulin, given his elevated Hemoglobin A1C. You write a prescription and book follow-up in 1 month.
