# Supplementary material for: “There’s no billing code for empathy” - Animated comics remind medical students of empathy: a qualitative study
Source: BMC Med Educ. 2016 Aug 12;16:204. doi: 10.1186/s12909-016-0724-z (PMC4983096; doi:10.1186/s12909-016-0724-z)
Supplement: Additional file 2: Semi-structured interview guide. — First version of semi-structured interview guide used for focus group interviews. (PDF 267 kb) [file 12909_2016_724_MOESM2_ESM.pdf]

Supplementary File 2. Semi-structured interview guide

Thank you all for participating in this study and coming in today.

You have already completed a baseline questionnaire, read over two comic strips, and completed another questionnaire.

The purpose of this session is to reflect on the comic strips and consider how they affect your interactions with patients.

I will ask a series of open-ended questions. Please keep in mind that there are no right or wrong answers. To help me with gathering and analyzing the results, I will be recording your responses. There won’t be any identifying information on these recordings. They will be kept for 5 years after publication, and then the files will be destroyed. The transcribed answers will be secured in a safe place without any identifying information on them.

Does anyone have any questions before we begin?

Impact of the Comics

1. What impact, if any, did reading the comics have on you?
    a. Prompt if needed: How did you feel while reading the comics?
2. Did the comics affect your approach to patient interactions? If so, how?
3. How do the comics compare to what you’ve learned so far in medical school?
    a. In terms of learning about chronic disease and communication skills
    b. In terms of method of learning

4. How do the comics compare to what you would do in a similar situation?
5. What would you do to improve the comics?
    a. to make them more effective in improving communication skills?
    b. to make them more engaging?
6. Any other final comments?
