# Supplementary material for: “There’s no billing code for empathy” - Animated comics remind medical students of empathy: a qualitative study
Source: BMC Med Educ. 2016 Aug 12;16:204. doi: 10.1186/s12909-016-0724-z (PMC4983096; doi:10.1186/s12909-016-0724-z)
Supplement: Additional file 3: Table S1. — Description of data: Characteristics of the study participants. (PDF 32 kb) [file 12909_2016_724_MOESM3_ESM.pdf]

Supplementary File 3

Table 1. Characteristics of the Study Participants

**Characteristic**	**n**	**%**
Age range		
21-25	22	88
26-30	3	12
Gender		
F	11	44
M	14	56
First language is English		
Yes	22	88
No	3	12
Medical school class		
1st year	13	52
2nd year	12	48
Highest prior education		
≥3 undergraduate years	20	80
Master's Degree	5	20

Undergraduate training		
Bachelor of science/health science/applied science	24	96
Bachelor of engineering	1	4
Medical specialty interest		
Family / Community Medicine	16	64
Dermatology / Ophthalmology	2	8
Emergency Medicine	6	24
Internal Medicine	19	76
Obstetrics & Gynecology	4	16
Pediatrics	10	40
Psychiatry	2	8
Radiology	3	12
Surgical specialties	9	36
Other	4	16
Anesthesia	1	4
Rural, wilderness	1	4
Preventative and public health	1	4

Not specified	1	4
Prior clinical experience		
No	24	96
Yes	1	4
Prior patient exposure		
<8 hours/month	6	24
8-12 hours/month	18	72
12-16 hours/month	1	4
