# Supplementary material for: “There’s no billing code for empathy” - Animated comics remind medical students of empathy: a qualitative study
Source: BMC Med Educ. 2016 Aug 12;16:204. doi: 10.1186/s12909-016-0724-z (PMC4983096; doi:10.1186/s12909-016-0724-z)
Supplement: Additional file 4: Table S2. — Description of data: Themes and representative quotes from qualitative data. (PDF 53 kb) [file 12909_2016_724_MOESM4_ESM.pdf]

Supplementary File 4

Table 2. Themes and representative quotes from qualitative data

Theme/subtheme	Representative Quote
**1. Empathy decline and its barriers**	
a) Awareness and concern of empathy decline	"It's a continuum of cynicism that continues to grow and develop and that negativity doesn't stop building…a simple act of kindness and care is perceived as something that's going to be weeded out eventually." (Participant 30, male, year 2)<br>"I have a fear that I will end up like that and…the way that people describe it, it's so inevitable. They're like just wait, it will happen to you too and it's like, what happens? How do you end up like that? Can I do something about it?" (Participant 27, male, year 2)
b) Barriers to maintaining empathy	
Environmental barriers	
i. Focus on medical expert	"Doctors are paid to be diagnosticians, not empathetic conveyors"<br>(Participant 6, male, year 2)

ii. Unhealthy environments	"How can I be empathetic with a patient when I walk out of the room and I get yelled at by a nurse for writing a note messy or get yelled at by my staff for not answering a certain question correctly…all of those things together have the potential to really strip an individual of their ability to be empathic." (Participant 30, male, year 2)
iii. Lack of financial incentive	"There's no billing code for empathy. And unfortunately for a lot of doctors, without that billing code, spending 15 minutes with that patient or spending an hour, they all get paid the same amount." (Participant 22, female, year 2)
iv. No academic consequence to lack of empathy	"If your technical skills are below the standard, there's consequence to you, but if you go through all the OSCE stations and you get most of the boxes, you're technically proficient but you're not a terribly empathetic person…there really isn't a consequence to you, you'll still filter all the way through all the levels of training and you end up in practice." (Participant 27, male, year 2)

v. Lack of positive role models	"I only had 2 or 3 patients under my "care". When I was dealing with these patients I would go and I would check up on them and ask hey how's it going, can we do anything for you, how are you feeling, can we make you more comfortable…And I went through that and my preceptor looked at me and said that's very cute…It was condescending, it was awful to hear and to experience." (Participant 30, male, year 2)
vi. Focus on passing examinations	"Maybe something is lacking in the training that it (empathy) tends to become just a checkbox, a cursory statement that's sufficient to pass the station." (Participant 20, male, year 1)
Student barriers	
vii. Limited time	"I think you're just trying so hard to keep up, you're probably using every bit of spare time you have to go look up stuff or keep running around to see more patients and you don't set aside the time cause you're probably stressed and just trying to stay on top of everything, let alone go that extra mile for the patients." (Participant 25, male, year 2)

viii. Personal stress and fatigue	"Clerkship, there's going be more hours, it's going to be more stressful and you're going to feel more lost…and I think that would also take away from your ability to be empathetic because you're kind of dealing with so much more stress yourself that it might be hard to put yourself in someone else's shoes to what is stressful to them." (Participant 28, female, year 2)
ix. Lack of social diversity	"Before…we were around a lot more people with a lot more diverse backgrounds but now we're in this stream where everyone's in medicine and you're always with them because it's so much of our life. So much of your way of thinking becomes concentrated…you kind of lose the ability to really relate to people who aren't in medicine." (Participant 26, female, year 2)
x. Desensitization	"If you have so many patients with diabetes, you're kind of accustomed to it and it's just something you treat and you tell everyone the regimen that you should put them on but it's easy to forget how they think about." (Participant 11, male, year 1)

c) Strategies to halt empathy decline	"Providing more feedback around it…how the patient felt and suggestions on what you could do next time or how something you said wasn't received well because it's one of those things that is hard to teach and hard to learn and also hard to know if you really are being empathic." (Participant 28, female, year 2)<br><br>"Validating people's concerns is a big one…because I think that when people feel heard, they feel like you actually cared. If you even paraphrase what they said back, that can take 2 seconds. I think that helps build a sense of you're listening to me and you think my concerns are valid. It might be that…if this were happening to you, you wouldn't be concerned about these particular things. But to this patient, that's what's important and just validating that, I see thatthis is important to you, is a quick way to include empathy into your daily interactions." (Participant 28, female, year 2)
2. **Impact of the comic and focus group on knowledge, attitude and skills**	
a) Comics shed light on the patient perspective and on chronic disease management (knowledge)	

i. Increased knowledge of the patient perspective	“Looking at something like this, which again brings it back to the context of the patient, it makes you understand their perspective and sort of puts you in their shoes”. (Participant 30, male, year 2)<br><br>“The comic really helped visualize and bring to life what exactly is going on instead of just words and listening to it …and really solidified what it actually means for a patient to go through a various routine cycle every single day for all the days of the year. I think actually seeing someone in the comic going through that really helped me fully realize what exactly is happening in the patient’s perspective”. (Participant 16, male, year 1)
ii. Drastic shift in knowledge	“I had the first one completely wrong, I kind of assumed that the patient would be relieved that they could just start taking the meds because they had trouble with their lifestyle…and they wouldn’t have to maybe put so much effort in the other areas…but it turned out it was the opposite of that, they were terrified of starting on insulin.” (Participant 25, male, year 2)

iii. Recognized difference between patient's perceived and actual perspective	"It can show two different things like how a patient looks on the outside but how he looks on the inside. So as a doctor, you may see a patient who might not look too worried when really inside his head, inside the bubble he's going crazy. Which I find that's one really good theme of this comic." (Participant 15, male, year 1)
iv. Recognized difference between patient and physician perspective	"In this slide, you can really see how the doctor, he probably gave more reasons why the patient should go on insulin but you see what the patient's really seeing and he's not really absorbing any of the reasons. It's just blah blahblahblahblah in his head and he's thinking something totally different." (Participant 19, female, year 1)
v. Increased knowledge of chronic disease management	"Having to take a pill however many times a day and checking your blood pressure every day like in the second comic. I guess I personally don't really think about how all those little things can be so annoying and really affects someone's quality, day to day life." (Participant 1, female, year 2)

vi. Recognized strategies used to motivate patients in self-management	"I think the importance of motivational interviewing, setting realistic goals, ensuring that the patient has strong support from someone (friend, spouse, whoever) and regular follow-up was well-depicted." (Participant 30, male, year 2)
b) Comics emphasized the importance of empathy in patient encounters (attitude)	
i. Value of understanding the patient perspective	"For example in the comics like understanding a patient's context and where they are coming from impacts how you perceive what their struggles are, what their level of motivation is, what their barriers are." (Participant 28, female, year 2)
ii. Effect on future patient encounter	"I will definitely try and take the extra few minutes where I can to ask how the patient is doing as a whole, and be sure to ask them what they're really concerned about, and how any news I might have for them is affecting them." (Participant 25, male, year 2)
c) Comics enhanced one's observational skills regarding communication (skills)	

i. Identifying non-verbal communication	“The doctor seems to be all business here, just giving him (the prescription)…he’s like here take this and go. If you compare this one here where he’s standing and giving the prescription to this one here, where he’s sitting down, that shows a big difference in how he’s communicating and how he’s trying to work with the patient. And you just seem more patient when you’re sitting down rather than standing up. Standing up is like here take this and get out of here so I can see the next patient.” (Participant 10, male, year 1)
ii. Identifying repetition	“I felt during the second comic especially, going through that daily slog, you just kept scrolling and scrolling and scrolling and things kept happening and he kept doing more”. (Participant 30, male, year 2)
iii. Identifying colour change	“I think the first couple ones it was pretty much mostly black and white…it really made the physician seem really impersonal or you felt there was a sense of coldness from him but then the next set you saw these colours, he kind of warmed up a bit. I think the colours really helped bring that out.” (Participant 5, male, year 2)
**3. Role of the comic in the curriculum as a reminder of empathy**	

a) A reminder of empathy	"It reminded me of empathy and the importance of that and brings you back to the point of view of the patient. It wasn't something new to me…it was something I've seen before but it was still a good reminder." (Participant 14, male, year 1)
b) Use during times of stress	"I think that's sort of a time when one of these comics would be really useful. You're really tired on internal medicine and you're having a crap day because somebody's yelled at you because you didn't do something right and then you sort of have to go see this next patient and you know you've been on call for 23 hours, last patient of the day, and it might be a nice time, pull out one of these comics, take a look at it, and sort of ground yourself and say you know what, it's kind of bad on the other side of this equation too." (Participant 22, female, year 2)
c) Use during clerkship	"This is something that we're kind of bombarded with first and second year, the emphasis is so much on communication. Maybe it could be more useful once people start to become more disillusioned in third and fourth year." (Participant 19, female, year 1)

d) Content specific to the clinical block	“They should have some relevance to whatever rotation you’re in. Like if I was on medicine and seeing a patient with diabetes, I think it would be very effective to actually have that whereas if you’re doing pediatrics and just trying to get a reminder about empathy, I don’t think it’s going to have the same effect. So you should tailor them to each block.” (Participant 25, male, year 2)
e) Use as a repeated reminder	“Now if I had variations of this with different comics shown to me at various stages in my month or year just as a reminder, it’s way more effective than if someone just told me, oh you have just got to be more empathic.” (Participant 11, male, year 1)
**4. Comics as an effective medium**	
a) Memorable	“Images are likely to stick in my head” (Participant 1, female, year 2)

b) Efficient	"They're short, so they get their point across in a very compact manner and I think that's something that's very important…you can read a textbook that's a 1000 pages long and get an idea of any kind of condition but then this presents a story in a very short format that's attractive and is still informative." (Participant 10, male, year 1)
c) Visual effects help personify characters	"Speaking to people on a media that's really rich, pictures are way more interesting and way more descriptive than words; and the graphics…especially for things that have to do with emotions, you could see the distress, the sadness, you were reading the picture for the emotions" (Participant 27, male, year 2)
d) Allows for reflection	"It also allows you to go at your own pace, as opposed to a movie, which just continues at a certain length. If you want to dwell and think about something that you've seen in the comic, it allows you to think about that." (Participant 8, male, year 2)
